# Patients experiences of self-management and strategies for dealing with chronic conditions in rural Malawi

**DOI:** 10.1371/journal.pone.0199977

**Published:** 2018-07-02

**Authors:** Vibian Angwenyi, Carolien Aantjes, Murphy Kajumi, Jeroen De Man, Bart Criel, Joske Bunders-Aelen

**Affiliations:** 1 Athena Institute for Research on Innovation and Communication in Health and Life Sciences, Faculty of Sciences, Vrije Universiteit, Amsterdam, The Netherlands; 2 Unit of Equity and Health, Department of Public Health, Institute of Tropical Medicine, Antwerp, Belgium; 3 Barcelona Institute for Global Health (ISGlobal), Hospital Clínic, University of Barcelona, Barcelona, Spain; 4 Health Economics and HIV/AIDS Research Division (HEARD), University of KwaZulu-Natal, Durban, South Africa; University of Antwerp, BELGIUM

## Abstract

**Background:**

The high burden of chronic communicable diseases such as HIV/AIDS, and an escalating rise of non-communicable diseases (NCDs) in Malawi and other sub-Saharan African countries, calls for a shift in how health care services are designed and delivered. Patient-centred care and patient self-management are critical elements in chronic care, and are advocated as universal strategies. In sub-Saharan Africa, there is need for more evidence around the practice of patient self-management, and how to best support patients with chronic conditions in the African context. Our study explored self-management practices of patients with different chronic conditions, and their strategies to overcome care challenges in a resource-constrained setting in Malawi.

**Methods:**

This is primarily a qualitative study, involving patients with different chronic conditions from one rural district in Malawi. Data are drawn from semi-structured questions of a survey with 129 patients (from the third of four-part data collection series), 14 in-depth interviews, and four focus-group discussions with patients (n = 31 respondents). A framework approach was used for qualitative analysis, and descriptive statistical analysis was performed on survey data.

**Results:**

Patients demonstrated ability to self-manage their conditions, though this varied between conditions, and was influenced by individual and external factors. Factors included: 1) ability to acquire appropriate disease knowledge; 2) poverty level; 3) the presence of support from family caregivers and community-based support initiatives; 4) the nature of one’s social relations; and 5) the ability to deal with stressors and stigma. NCD and HIV comorbid patients were more disadvantaged in their access to care, as they experienced frequent drug stock-outs and incurred additional costs when referred. These barriers contributed to delayed care, poorer treatment adherence, and likelihood of poorer treatment outcomes. Patients proved resourceful and made adjustments in the face of (multiple) care challenges.

**Conclusion:**

Our findings complement other research on self-management experiences in chronically ill patients with its analysis on factors and barriers that influence patient self-management capacity in a resource-constrained setting. We recommend expanding current peer-patient and support group initiatives to patients with NCDs, and further investments in the decentralisation of integrated health services to primary care level in Malawi.

## Background

Malawi, like other sub-Saharan African countries, is experiencing a rise in chronic non-communicable diseases in addition to an already high HIV/AIDS burden [1, 2]. Malawi’s adult HIV prevalence in 2016 was estimated at 10.6% [3], while in a 2009 nationwide survey (n = 5206) assessing risk factors for NCDs, over 30% of adults were reported to have hypertension, and 8.9% had cardiovascular diseases [4]. This double burden of chronic communicable and non-communicable diseases requires a reorientation of national health care systems designed around an infectious disease-centred model [5, 6]. The concepts of patient centered-care and patient self-management are gaining global attention in the design and delivery of chronic care, and as proposed in international frameworks of chronic care [7, 8]. The core principles advocated in these frameworks are self-reliance and empowerment, which represents a shift from a traditional passive patient role in healthcare, to pro-active engagement of the patient in decisions related to his/her own health [9].

Self-management refers to the day-to-day tasks that a patient undertakes to monitor and manage condition-related symptoms, adhere to treatment and adapt to a healthy lifestyle, in order to achieve overall well-being and a satisfying life [9, 10]. Patients depend on the support from their health providers and caregivers, to make decisions and facilitate adjustments in their health behaviour [9, 11]. Bandura’s social cognitive theory describes that a patient’s ability to adjust is influenced by the interaction between their cognitive capacity (i.e mechanism to self-regualte, self-reflect and self-motiviate), their behaviour, and surrounding environment [12]. Self-management does not occur in isolation, but unfolds in a continuum whereby availability of resources, organisation of health care services, patient characteristics and health status also influence a patient’s ability and motivation to self-manage [13].

While self-management is a relatively ‘new’ concept for sub-Saharan African health systems, health care systems in high-income countries have well-established self-management programmes in place [11, 14]. Most African health systems currently find themselves in a transitional phase, whereby disease-specific services are modified into integrated health services for patients with chronic conditions [15–17]. The experiences gained over the years with HIV treatment and care at primary level largely influence this transitional process [6, 16, 18]. For example, during the HIV treatment scale-up, South Africa started introducing chronic care clubs and drug pick-up points at community level. These interventions target patients stable on treatment and are a means to decongest health facilities, and reduce the burden of frequent health visits for patients [19, 20]. More recently, Malawi introduced integrated chronic care clinics for patients with HIV and NCDs at secondary-level facilities, in a few districts [21]. This ‘one-stop’ clinic offers patients specialist care, where patients receive diagnostic and clinical consultation, adherence counselling, labatory and pharmaceutical services [21]. To ensure continuity of care at household and community levels in Malawi, community-based caregivers, expert patients and patient support groups are instrumental in offering services such as health promotion, adherence counselling, home nursing, home tracing and referrals, psychosocial support and livelihood support [22–26].

There is a growing body of evidence from sub-Saharan Africa which documents how patients with chronic conditions—the majority of studies focussing on HIV—self-manage their conditions with the support of health care teams, family and community caregivers. These studies have highlighted the importance of patient access to peer-support groups in the context of severe shortages in human resources for health, as a way to increase opportunities for patient education and support [27, 28]. Studies also point to the need for family caregiver knowledge on the patient’s condition, to help them make informed decisions together with the patient on issues of treatment adherence, symptom monitoring and daily adaptations within the home environment [29, 30]. Well-known health system challenges in sSA such as drug stock-outs, inadequately trained health professionals and poor health worker distribution, the cost of care and geographical access, have been cited as barriers in the delivery of chronic care and promotion of self-management [31]. Other studies have elaborated on patient-level challenges, resulting from economic hardship, which affect a patient’s ability to afford an appropriate diet and medication [28, 30–32].

As sub-Saharan African health systems are working towards incorporating the management of NCDs in routine health care services, there is need for further understanding of how concepts of patient self-management are currently being contextualised in these settings. Furthermore, there is need to expand the study focus on one disease, mainly HIV, to research that stretches across different chronic conditions and compares experiences and variations in practice. To further unpack self-management mechanisms and patient adjustment, we make use of Bandura’s social cognitive theory [12]. The aim of this paper is to explore self-management experiences and practices of patients with different chronic conditions in Malawi, and patient strategies to deal with care challenges. Generated evidence could be used to inform health providers, managers and programme implementers, responsible for the design of responsive chronic care services and support systems in sSA. This paper presents data from a research in rural Malawi, which includes semi-structured survey questions, interviews, and focus-group discussions with patients with different chronic conditions.

## Methods

### Study context

This study was conducted in Phalombe District, situated south-east of Malawi and near the border of Mozambique. Phalombe has a population of 393,587 people, and the major ethnic groups are the Lomwes (80%), the Mang’anja (15%), and the Yao (3%) [33]. The Lomwe group originates from northern Mozambique, and their migration, cultural integration and settlement in the southern districts of Malawi dates back to the 1900s [34]. The district is characterised by high poverty and unemployment levels, and the main economic activity is farming and small-scale trading [33].

In Malawi, chronic care services are provided through a three-tier structure of primary, secondary and tertiary health facilities that are linked through a referral system [1]. Due to the absence of a government-owned hospital, secondary care is provided in a district government health centre, and referrals largely depend on a Roman Catholic mission hospital [33]. The rest of the district is served by 11 health centres, two dispensaries, and one maternity unit [33]. Services such as clinical consultation, medication, diagnostics and ambulatory services are fully subsidised (free-for-user) in public health facilities, while private health facilities—the majority run by the Christian Health Association of Malawi (CHAM)—operate on a user-fee policy [1]. To support patients with their self-management practices, patient education and counselling is mostly done in clinic settings by health providers, and in HIV programmes, expert patients and patient support groups are engaged in this role [23]. Community home-based care programmes run by lay volunteers in community/faith-based organisations (CBO/FBOs) provide a link between patients, families and health facilities [25]. These community-based initiatives offer a wide range of support to chronically ill-patients including home nursing, health promotion, adherence counselling, and psychosocial support, which are critical in patient self-management practices [25].

### Study design

The paper primarily reports on qualitative data obtained from a research examining local models for chronic care and self-management support initiatives in Phalombe district, Malawi (2015–2018). Additional information can be accesed in S1 File. The research consisted of a longitudinal survey, interviews, and focus-group discussions with patients with different chronic conditions. The survey comprised of 140 patients with different chronic conditions (HIV and NCDs), and who were enrolled in community home-based care programmes. The aim of the survey was to evaluate patient self-management outcomes (health status, self-management behaviour, and self-efficacy), and the benefits of community-based self-mangement support initiatives. The survey was administed at baseline, and repeated after months three, six and twelve, using adapted scales from the chronic disease self-management programme (CDSMP) questionnaire [35, 36], (see also S1 File). In addition to the survey, we conducted interviews and focus-group discussions with a separate group of patients from the same catchment areas. The aims of these engagements were to deepen our exploration on self-management, and patient experiences and capacity in the Malawian context.

This paper presents findings from semi-structured questions included in the third survey round (month six), as well as interviews and focus group discussions (FGDs) with patients. Data were collected between April 2016 and April 2017 by the first author (VA), and three Malawi trained research assistants, although not from the same study district.

### Study population, sampling and recruitment

Survey and interview participants were patients recruited from villages across the catchment areas of five community/faith-based organisations (CBO/FBOs), in Phalombe district. The chronic conditions of interest were non-communicable diseases (e.g. hypertension, diabetes, epilepsy, asthma, stroke, and cancer) and communicable conditions such as HIV and tuberculosis. Survey inclusion criteria were adult patients (18 years and above), with one/more chronic conditions, who were newly registered into a CBO/FBO. In total, 140 patients were recruited from the five CBO/FBOs at baseline. In the third survey round (month six), we followed-up and interviewed 129 patients of the initial total, with 11 patients dropping out at this point (6 patients had moved outside the study area, 3 died, and 2 withdrew from the survey).

Interview participants were selected purposively, using the maximum variation technique [37], to represent diversity in patient’s characteristics such as gender, age, chronic conditions, presence of comorbidities, and geographic location. These patients were selected from registers of the same CBO/FBOs. We conducted interviews with 10 patients (5 females, 5 males) and 4 HIV expert-patients (1 female, 3 males). We organised four-mixed gender FGDs with approximately 7–10 patients in each group (20 females and 11 male patients).

### Data collection and tools

The survey was interviewer-administered using an electronically programmed application (Open Data Kit) which was uploaded to a secure web-based database. The semi-structured questions included in the third survey round explored self-management aspects such as: patient utilisation of health care; medication practises and care seeking experiences; resources and support to manage patient conditions; challenges and coping strategies. These questions were still rudimentary in the previous survey rounds. The longitudinal nature of the survey and frequent visits of the researchers to the participants’ homes, allowed for building of trust and opening up of the participants to the research team, and contributing to the expansion of semi-structured questions in the survey. Throughout the survey, the research team kept a journal on these home visits, which kept track of patient illness experiences and documented changes observed and incidents reported by the patients or researchers. We used these notes for further contextualisation of the survey results.

The interviews and FGDs were designed to run concurrently with the survey, allowing for the inclusion of emergent issues from the survey, as well as incorporation of self-management themes as cited in other literature [13, 38, 39]. Topic guides themes included: patient illness experiences; medication and care seeking practices; diet and lifestyle modification; managing work-life, family, social relationships and emotional wellbeing; patient support structure; and a cross-cutting theme that explored how patients managed challenges related to the above themes.

CBO/FBO officials assisted the research team to book appointments with patients meeting the study eligibility criteria. Discussions were held in respondent’s home or at a preferred location, and were conducted in the local language *(Chichewa)*. Audio recorded interviews were transcribed and translated to English. All personal identifiers in interviews and questionnaires were replaced with codes, and data from this research was kept securely and only accessible to research team members. Preliminary results were shared, and discussed with district and community stakeholders engaged in the study, as part of the validation process.

### Data analysis

Descriptive statistics of survey data were performed in STATA (Version 13; STATA Corp, College Station, TX, USA). Given the heterogenous nature of the patient population, we chose to group our analysis by disease category, that is, all HIV (with/out comorbidities) examined together, and non-HIV patients grouped together. This decision was informed by the observation of differences in health service provision for patients with HIV, and those with NCDs, and the potential influence of this difference on a patient’s perception of illness experiences and self-management.

All qualitative data (IDIs, FGDs and survey journal reflections) were managed using NVivo (Version 11, QSR international). Qualitative data analysis was performed using a framework approach, as described by Ritchie and Spice [40]. The first step involved developing an initial coding framework (by VA and CA). Codes were generated *inductively* through in-depth reading of transcripts, and *deductively* by drawing on questions included in interview guides. The coding framework was continuously expanded to capture emerging themes. After coding all data in NVivo, themes related to a particular concept were grouped together to form categories and exported to a word-text processor to generate charts/matrices. Charts were used to summarise data, identify similarities or differences, and explore patterns (e.g. among HIV and non-HIV patients) in the analysed data.

### Ethical considerations

Ethical approval was obtained from the Vrije Universiteit Amsterdam-Netherlands (EMGO^+^; WC2015-080, 27-Oct-2015), and the National Committee on Research in the Social Sciences and Humanities, Malawi (P.11/15/64, 10-Dec-2015). The Ministry of Health (Malawi), Phalombe district health office, the Archdiocese of Blantyre Catholic Health Commission, local leaders, and the participating CBO/FBOs gave consent for implementation of this research. Written informed consent was obtained from all respondents.

## Results

### Study participants characteristics

We present data from 129 survey patients, 14 in-depth patient interviews (four with HIV expert-patients) and four focused-group discussions (n = 31 patients)—see Table 1. The median age of interview and survey participants was above 40 years (range; 20–84), and over 70% of all participants were females. Among the 129 survey patients, 60.5% had HIV, 10% had HIV with other comorbidities, and 29.5% had a non-communicable disease. The most common NCDs among survey patients were hypertension (25.6%), epilepsy (7.8%), and asthma (3.88), while a few patients had cancer, diabetes or other cardiovascular conditions. Among the patients interviewed and FGD participants, 29 had HIV, of which 11 had HIV with other NCDs, and 16 patients had a non-communicable disease. Over half of the participants had less than five years of formal education. The majority of participants depended on subsistence farming or small-scale trading as their main source of livelihood.

**Table 1:** Characteristics of study participants.

DEMOGRAPHIC CHARACTERISTICS	Survey	Patient IDIs	HIV expert patient IDIs	Patient FGDs
TOTAL	129 (%)	10	4	4 (n = 31)
**Median age (range)**	42 (20–84)	42 (35–70)	43 (33–47)	54 (29–73)
**Gender**	34 (26.36) males 95 (73.64) females	5 males 5 females	3 males 1 females	11 males 20 females
**Patient conditions**				
HIV (all)	91 (70.5)	6	4	19
*1) HIV only*	78 (60.46)	5	2	11
*2) HIV with comorbidities*	13 (10.1)	1	2	8
Hypertension	33 (25.58)	1	0	5
Epilepsy & other mental health	10 (7.75)	1	0	2
Asthma	5 (3.88)	0	0	4
Stroke	4 (3.1)	1	0	0
Diabetes	3 (2.33)	1	0	0
Cancer	2 (1.55)	0	0	1
Heart condition	1 (0.78)	0	0	0
**No. of chronic conditions**				
1 condition	110 (85.27)	6	2	22
2 conditions	16 (12.4)	4	2	8
3 conditions	3 (2.33)	0	0	1
**Education**				
*No schooling*	15 (11.61)	2	0	3
*1–5 years primary school*	69 (53.49)	2	0	20
*6–8 years primary school*	35 (27.13)	4	2	8
*Secondary in/complete*	9 (6.98)	1	2	0
*College/tertiary and above*	1 (0.08)	1	0	0
**Main occupation**				
*Subsistence farmer*	60 (46.52)	5	0	9
*Casual labourer/trader*	43 (33.34)	2	0	15
*Public/private sector employee*	3 (2.33)	0	4	0
*Unemployed (unable to work)*	13 (10.08)	2	0	0
*Other (e*.*g*. *domestic worker)*	10 (7.76)	1	0	7
**CBO/FBO site and participants**				
FBO A	57 (44.19)	2	1	2 (14)
CBO B	25 (19.38)	2	1	1 (7)
CBO C	25 (19.38)	2	0	0
CBO D	22 (17.05)	2	1	1 (10)
FBO E	0	2	1	0

The first part of the results will examine patient health and care experiences, while the second part discusses patients’ adjustments and self-management practices.

### Patient illness experiences

Patients reported they often reflected on their past and present health, portraying the dynamics that took place in their lives due to their conditions, and how these impacted in the way they took care of themselves. For instance, patients discussed how they experienced symptoms (like pain, fatigue, and body weakness); the impact of their condition and medication on their physiology; actions taken and recovery process—illustrations in Table 2.

**Table 2:** Patient illness experiences quotes.

Patient category	Examples
HIV only	**IDI06_male_HIV**: ‘*I am not feeling well since the start of my illness…right now I just feel stiffness in my legs*, *if I just take off my shoes I cannot walk* …*when I also take medicine eh*! *it did not go well with the first medication*, *my breasts were enlarged as if I want to breastfeed*, *so they [health workers] changed [medication] for me when I went and complained*.*’*
	**IDI09_female_HIV**: ‘*Right now I can differentiate with how I was in the past…it shows that things are changing…diet is what seems to be a problem*. *I also have body pains…’*
	**KII08_male_HIV-EP**: *‘I was taking 5A [HIV regimen] and because of that my breasts started like swelling*, *so I told the doctor and they changed the medication but now…if I walk without shoes I feel like my feet are very hot…When I face a problem that concerns medication*, *I go see the doctors* …*’*
HIV with NCD	**FGD02_patients_A**: *P2*: *‘…when I have taken my medicine…I don’t see any problems*. *I can even stay for months without having any problems doing my work*. *But if I miss even for just a single day…sometimes I fall unexpectedly’* ***(female*, *HIV + Epilepsy)***
	**S132_male_HIV+Stroke**: Patient experiencing paralysis of his left side of the body. Relies on his wife to collect antiretroviral drugs from hospital. Not on any stroke medication. Advised by health worker to exercise his hand while at home; he carries eight bricks a day and helps with farm work. At present, he feels his stroke condition is not improving.
NCD only	**IDI07_female_DM+HBP**: ‘…*I just started experiencing signs like feeling itchy…*.*and I could urinate during the night for maybe fifteen times…sometimes when I wanted to walk*, *my legs would feel heavy*. *So at that point I went to* …*a health centre…when they tested me*, *they found that I had too much sugar [blood glucose]’*.
	**IDI04_male_Epilepsy**: *‘…when I wake up my heart beats a lot [and] when I laydown* …*it takes time for me to have an attack [seizure]…they [people] say that this illness has its own time*, *they [people] say that when the moon is on this side it [seizure] happens*, *but when you are quick enough to take medicine*, *it does not happen*. *It’s like you have blocked it with the medication*. *Instead of you falling down*, *you don’t*.*’*
	**S012_male_HBP**: Stopped taking anti-hypertensive drugs, he says his doctor advised his condition had stabilised. When he walks long distances, he feels dizzy, shivers a lot and sometimes falls down. Takes paracetamol when he feels pain.

In HIV patients, the knowledge of their sero-status facilitated the initiation to care and treatment, which in turn led to overall health improvement and increased independence, as illustrated in the quote below;

…*the time when I did not know of my status…I was very sick not expecting to be alive up to date like the way I am…I was not able to eat*, *to talk to anyone*, *or even to sit like this*, *no*! *I could only sleep and maybe do my toilet right where I was*. *But just after I started taking my medicine…I see that I am getting much better…*

(***IDI02_Female_HIV_D)***

In non-HIV patients, recognition and cues to action tended to be a longer process. For instance, some hypertensive and diabetic patients discussed how they struggled with prolonged symptoms before getting medical attention, partly due to their unawareness of signs and symptoms associated with their condition.

A concern among patients with multiple conditions was how medication inconsistency impacted on their overall health, and their inability to manage complications resulting from different conditions. In the absence of medication, some patients persevered without action until their symptoms subsided. A few patients demonstrated learning how to manage their conditions through minimizing potential risks that could trigger worsening of their condition. For instance, an epileptic patient reported that in an effort to control his seizures, he avoids being near fires, crowds and heights.

### Experiences with medication and health care

Patients reported being aware of the need to be on lifelong treatment, although several factors limited their ability to do so. To investigate this further, we explored self-reported medication adherence in our survey. Almost 40% of the patients in the non-HIV group indicated they had missed their medication in the past 30 days, compared to 20% of those in the HIV group (*p<0*.*01)*—see Table 3. One barrier reported was the availability of medication, particularly in the public health sector where essential drugs were not consistently available. In this context, almost 70% of the survey patients relied on public health facilities as their primary point of care. Reportedly, anti-epileptic and anti-hypertensive drugs were frequently out of stock, and cancer and stroke medication were reported to be mainly accessible in referral health facilities, requiring patients to travel and incur additional costs.

**Table 3:** Medication and health care seeking practices.

Medication and care seeking practices	HIV group *n = 91 (%)*	Non-HIV *n = 38 (%)*	Total *n = 129 (%)*	Chi-square (p-value) *HIV vs non-HIV group*
*Whether missed medication in past 30 days*	18 (20)	15 (39.47)	33 (25.58)	6.69 (0.01)
*Number of days missed medication*				
<5 days	11	8	19	
5–9 days	3	2	5	
10–14 days	0	1	1	
14–30 days	0	2	2	
>30 days	4	2	6	
*Where patients access care*				
Public PHC	40 (43.95)	14 (36.84)	54 (41.86)	
District public referral health centre	18 (19.78)	17 (44.74)	35 (27.13)	
CHAM PHC	10 (10.99)	3 (7.89)	13 (10.08)	
District mission hospital/other tertiary	22 (24.18)	2 (5.26)	24 (18.6)	
Not seeking facility care	1 (1.1)	2 (5.26)	3 (2.33)	
Whether patient use traditional *medicine for their condition*	22 (24.81)	10 (26.32)	32 (24.81)	0.07 (0.8)

In efforts to deal with these challenges, some patients modified medication schedules either by saving drugs for future illness episodes, or only taking drugs when experiencing symptoms. This seemed to be a particular popular strategy among hypertensive patients. A ‘cost-saving’ strategy in patients residing in the district headquarter catchment area consisted of patients only visiting the district mission hospital for HIV and tuberculosis care (which was free of charge), and switching to the district public health centre, for general and specialised care (which were not free of charge in the district mission hospital). In the more distant areas near the Malawi-Mozambique border, patients reported they could not afford care in the private/CHAM health centres in their catchment area, and some patients would cross over to purchase medication from drug vendors in Mozambique.

We also explored whether patients tried other alternative treatment to manage their conditions. Some patients tried using herbal/traditional medicine at the onset of their condition but discontinued when they found it to be ineffective. Other patients used them consistently when they discovered drugs were unavailable in their primary point of care. Some patients tried these treatments when they experienced drug reactions or persistent symptoms, which they felt were not adequately managed by providers in the public health sector—additional examples in Table 4.

…*I was not at peace…early in the morning I find myself just scratching, because it [my body] is very itchy…since I went to the hospital…the medicine they gave me I applied but did not help…So someone told me that they think this is a big illness, some [people] said it was leprosy, so I went and looked for traditional medicine and that is what I take*…

**(IDI08_Female_Patient_HIV_A)**

**Table 4:** Medication and health care experiences quotes.

Patient condition	Examples
HIV only	**IDI02_female_HIV**: *‘…If I hire a bicycle I pay 2500 kwachas [approximately 3 euros] when going [to the clinic]…if I have no money for transport I leave very early in the morning around 3 o’clock’*.
	**IDI05_male_HIV**: *‘…when I drink my medicine and I have not eaten*, *I experience some problems…my body becomes weak and I feel confused…I feel as if I am drank…’*
HIV with NCD	**SP10_female_HIV+Ulcers+HB**: Regularly takes her HIV drugs. Stopped taking anti-hypertensive drugs due to frequent urination. Used to take honey to manage ulcers and believes that her ulcer is ‘cured’.
	**IDI01_male_HIV+Cancer**: *‘…to get my weekly medicine [cancer] I am supposed to go there [hospital] every Tuesday*. *So if there is no money…I have to borrow*.
NCD only	**IDI07_female_DM+HBP**: ‘*The drugs [for diabetes] I am ok with have ended two days ago*, *so I am just taking the other [new prescription] medicine…which I received some two months ago and I just kept [them] in order to finish the ones I am ok with*. *With the other [new] medicine*, *I was just trying it out*, *but I have seen that there is no problem*. *I have started taking these new drugs two days ago*.
	**S079_female_DM+HBP**: Collects her medicine from two referral facilities; anti-hypertensive drugs from a public health facility, and diabetes drugs from a private health facility. She has her doctor’s number, who usually calls to notify her to collect her diabetic medication before running out.

Experiences of symptoms and side effects also influenced treatment inconsistencies and modification. For instance, some hypertensive patients stopped taking their medication when experiencing side effects whenever they took their medication, while others discontinued taking their medication regularly, if they had no condition-related symptoms. In HIV patients, some skipped their medication if they did not have a meal, explaining that taking medication on an empty stomach made them drowsy.

Another challenge, especially reported by HIV patients, was how their social environment and people’s awareness of their HIV status, made them feel uncomfortable and influenced their choices of where to seek treatment and care. Some patients went to distant health facilities to avoid being seen by neighbours or other community members. Some kept their patient-held records (referred to as health passports) out of reach from their spouses and other family members, while others possessed more than one health passport to access health care services i.e. a separate one for HIV and another for general conditions, as a way of concealing their medical history.

…*A lot of people [patients] are failing to receive appropriate help because they are afraid. Others [patients] have reached a point where…they are hiding drugs so that their spouse does not see…which is not helpful because when you follow what the doctor tells you…you have a good life. But when you are hiding, sometimes when you go to the hospital because you are afraid, you find people you know and you say today I am not receiving medicine, and maybe in your bottle there was only one pill remaining*…

**(IDI01_Male_HIV+Cancer_D)**

### Dealing with adjustments to a life with chronic conditions

#### Dealing with everyday needs, roles and relationships

The desire for maintaining a stable health, an ability to pursue everyday activities, and to live longer were cited as the main aspirations by patients with chronic conditions. Most patients reported of how condition-related pain and fatigue limited their ability to perform daily chores including farming and domestic work. Patients with mobility challenges (such as stroke, cancer and epilepsy) required extra help with personal care such as bathing, wound care, help with getting to the toilet and into bed, and relied on family caregivers to take over activities that were strenuous or labour-intensive for them.

Families were instrumental in the way patients managed their conditions and made adjustments. We explored, in survey and interviews, the nature of family support and how these relationships were characterised. Some patients described receiving support (such as food, financial assistance, emotional encouragement and counsel, assistance with medication and clinic visits) from immediate family members and relatives living within same households. A concern raised was how frequent illness episodes and care demands weighed on family caregivers, hence their support became infrequent (due to other responsibilities or resource constraints), or discontinued (if caregivers resided further away or felt overwhelmed)–additional examples in Table 5.

When I was critically ill, [my elder brother] was really helping. But when he saw that I started recovering…he stopped giving money …but I still explained to him that I am not fully recovered, so he should still continue assisting otherwise I will die…since that time, I don’t argue with him because [he has] cared for me for a long time

**(IDI05_Male_HIV_E)**

**Table 5:** Illness, work and livelihood quotes.

Patient category	Examples
HIV only	**IDI06_male_HIV**: ‘*I*: *In what ways does this illness hinder you from the activities you want to do*? *P*: *Not having the strength to farm*, *and with the [body] stiffness*, *not even seeing her [my wife] as a woman*, *I just look at her*. *I*: *You just look at her*? *P*: *Yes*, *I just look at her*. *I do nothing*, *just as long as she is cooking for me*.*’*
	**IDI08_female_HIV**: *‘…these days if I get a hoe to farm*, *I start feeling breathless…I come back [home] and sleep*. *So this year I have not farmed…in the past years*, *I would have a garden of vegetables that I would sell*, *but these two years I have not done that*, *so it has been hard…’*
HIV with NCD	**KII10_female_HIV+Asthma-EP**: ‘*So we [in support groups] discuss what we can do so that we live a healthy life*. *What type of food we need to eat so we can stay as healthy people*, *and how we can find these food*. *Other [support group members] may come with their worries of how they cannot find food because they are positive [HIV]*. *So we encourage one another that even though we are [HIV] positive*, *we should not look up to others for help*, *but we should start ourselves…maybe through farming’*
NCD only	**IDI07_female_DM+HBP**: *‘I was told [by a health worker] that when I go to the farm I should plough small portions*, *maybe dig a few ridges because I don’t have the strength to do [farming]*.
	**S023_male_HBP**: Patient finds it difficult to moderate salt and oil in his diet, since his granddaughter prepares meals.
	**FGD02_Patients_A** *P2*: *´…at the hospital they say that I should not be very close to fire*, *avoid going to the river to wash and walking in places where no one can go…If I go to collect firewood*, *that I should not do that frequently…it is hard for me to follow these instructions*. *I try as much as possible to push myself to go to the mountain…I get firewood*, *sell it*, *and buy salt and soap for my children´* ***(female_epilepsy)***.

Patients in marital or cohabiting relations narrated of how their conditions impacted on their relationships. Patients who were in steady relationships praised the support and care received from their partners. On the other hand, there were patients who experienced problems in their relationships. For instance, HIV patients narrated of how disclosing their health status to their partners had resulted into separation (experienced by both male and female patients). For female HIV patients choosing to remarry, there were reports of verbal insults from new male partners, when they learnt of their HIV status, while some reported their partners threatened to leave their homes when there were disputes—in this setting, as part of matrilineal customs, men mary and could settle in their wife’s maternal home. Among elderly patients, some indicated when they developed chronic illnesses, the affection by their spouses subsided over time, resulting to feelings of abandonment.

…*in my house…there are about seven children alive, three died…some time back we had an argument in the house; so the argument was between the two of us [patient and wife] of why we are not having intercourse. So I asked her ‘is it because I have this illness?’, and she said yes but in my heart I just said “okay, no problem”* …

**(IDI04_Male_Epilepsy_B)**

#### Diet and lifestyle adjustments

Patients recognised the importance of a proper and balanced diet, and this information was reinforced to them in their interactions with health providers. Hypertensive and diabetic patients reported being aware of certain food restrictions and diet modifications, for instance low salt, oil and sugar consumption. HIV patients often discussed the importance of having a nutritious diet that was locally available, and as taught by their health providers, and in patient support groups. There was a general awareness in patients of the need to minimize tobacco and alcohol consumption.

Patients reported facing challenges in adhering to recommended diets as a result of food shortages and a lack of household income. Their large dependence on agricultural yields and seasonality for the availability of for instance fruits, were major determinants in the diet composition of patients. Subsequently, patients had no other option than to have suboptimal food portions, skip meals or stay for days without a meal, which as discussed earlier, affected their medication intake. Patients attempted to overcome these challenges by looking for paid piece-meal work or selling their farm produce to obtain money for buying food. HIV patients spoke of programmes for patients with nutritional problems in their health facilities, in which they tried to enrol if they experienced food shortages. The moderation of salt and oil intake in hypertensive and diabetic patients proved to be challenging and depended, among others, on the adherence of their caregivers when food was prepared for them—additional examples in Table 5.

With regards to observing physical wellness, patients walked for long distances as well as cycled, although these activities appeared to be instigated by the poor availability of public transport and road infrastructure in the district, rather than the need for healthy exercise. Patients also engaged in physical work such as farming, collecting firewood and drawing water, which were perceived as physical exercise. For patients with mobility challenges and physical disabilities resulting from their chronic conditions (e.g. stroke, diabetic and some epileptic patients), there was little health advice and guidance offered from health providers on how this group of patients could best engage in healthy exercise.

#### Psychosocial adjustments: Dealing with emotional distress and stress management

Chronically ill patients experience emotional distress due to the disease itself, or due to different stressors from their external environment such as difficult social interactions, which could lead to patients feeling depressed, anxious, or frustrated. We tried to explore whether patients with chronic conditions felt or experienced differential treatment from others because of their conditions. Both HIV and NCD patients (especially epilepsy, stroke, and cancer) narrated experiences of being verbally abused, ill-talked about by community members or even being isolated from social and community activities. Some HIV patients were not considered in social welfare projects or development projects as eligible beneficiaries—see examples in Table 6.

**Table 6:** Dealing with stressors quotes.

Patient category	Examples
HIV only	**IDI05_male_HIV**: *P*: *‘…when I was very sick*, *my [relatives] said “you AIDS person*, *is this not AIDS*? *why are you not dying*?*”* *I*: *Why do you think people say such things*? *P*: *…lack of knowledge and ignorance is what is making them [people] to say such things*. *But they don’t know what is coming ahead of them*.*’*
	**KII02_male_HIV-EP**: *‘…The problems are being gossiped by people and being insulted…there are some friends who think of themselves as good [noble]*, *who insult us [HIV patients] and discriminate us on certain things [activities]…in the village*, *you could be at a place and you just notice from people’s faces that maybe people are not happy with your presence…you just find yourself moving away from them so that they stay alone…’*
HIV with NCD	**IDI01_male_HIV+Cancer**: *‘…being together with people to share the word of God*, *it brings encouragement especially when you hear testimonies from others who have been healed by Jesus from similar diseases*. *You feel encouraged that God can heal me too*. *But since it is said that the wages of sin is death*, *I have accepted on my part that maybe this is the prize for the things that I used to do in the past*. *When I meet such people*, *we always pray to God for forgiveness*.*’*
	**FGD01_Patients_C**: *P1*: *‘…I planted a guava tree at home and people were saying you will not eat the guava [fruits] because you have the disease [HIV]*, *but I said no*! *Now the tree is bearing fruits and I am eating the fruits…’* ***(female*, *HIV+Cancer)***
NCD only	**IDI04_male_Epilepsy**: *´…my friend from Mozambique*, *when he came and passed by a nearby house*, *he just heard that I am now epileptic…he did not even bother to come and see me*, *he turned back…maybe he was afraid he would get the disease if he comes to see me*.*’*
	**IDI07_female_DM+HBP**: *P*: *‘I really have problems; I worry and ask what was God thinking for me to have such diseases*. *I*: *So how do you deal with the problems that you have*? *P*: *Sometimes I pray and just say anyway*! *and then I just forget about everything and stay as if I don’t have any disease’*

Patients’ ways of dealing with such challenges were self-isolation, avoidance of situations or people contributing to these negative experiences. Some reported such incidents to their local authority, where in some locations they had local by-laws penalizing acts of reported HIV stigma/discrimination. Some patients preferred sharing these experiences with significant others or with their health providers. Some of the HIV patients felt patient-support groups provided a ‘safe space’ and emotional support to deal with such experiences.

…*we [patients in support groups] meet and we encourage one another that we should not be worried… even the people that could be discriminating us today, tomorrow they will join us. So we are not worried about that because we accepted and made the decision to come out in the open after we had tested*…

**(KII10_Female_HIV+Asthma_E)**

Some HIV patients, whenever they experienced negative sentiments from others, neutralised such situations by projecting strength, hope, making social comparisons and showing a sense of pride in knowing their HIV status, and in taking charge of their future.

…*I don’t get anxious that people are laughing at me, because I know that those laughing at me do not know of their future, while I know mine*…

(**IDI02_Female_HIV_D)**

Patients reported that uncertainties about their future life and of their dependents were emotions that led to the development of anxiety. In the event of stress, patients engaged in different activities, which for most patients was either praying or attending spiritual gatherings, to help them cope with their condition and life challenges. Others visited family or friends for a chat, and some reported sleeping helped them to relax. Some patients engaged in household chores or other forms of work to direct their negative thoughts. HIV patients wanted to feel ‘normal’ or strived to do things that were considered to be part of communion with others such as ‘eating from the same plate’ and maintaining friendship—additional examples in Table 6.

## Discussion

Our paper provides insight into the lived experiences of patients with chronic conditions in a resource-constrained setting. An important finding was that patients demonstrated capability to manage their conditions, given this context. Our findings concur with other studies [13, 28, 32], which discuss several of the individual and contextual factors that influence self-mangement practices. Furthermore, these studies and our findings show that patient’ adaptiveness is an individualised process and relies on how well a patient is able to cope with the circumstances in their environment. Bandura’s social cognitive theory further explains adaptation as a process whereby patients develop different mechanisms [12]. For instance, patients create internal models based on successful management of past experiences/events to deal with current challenges (symbolising); or choose to avoid situations that cause distress (self-regulation); or observe and learn complex skills from others such as peers in support groups (vicarious learning) [12]. We found evidence of the development of such mechanisms, but it proved difficult to delineate them from the broader patient environment, which was characterised by a heavy reliance on family support and (in)access to resources in their external environment. This heavy reliance, often resulting from economic hardship and insuffient health care coverage, profoundly shaped patient experiences and strategies for self-management.

While HIV patients were able to access most of their health care needs at primary care level, with constant availability of antiretroviral treatment, patients with NCDs (including HIV comorbid patients) experienced difficulties, and had to seek services beyond primary level and public provision. A possible explanation for the difference in care experiences could be a reflection of the extensive support and resource allocation towards the advancement of HIV services in Malawi [41]. The cost-implication of being referred to secondary or tertiary level care for diagnostics, consultation, medication or even disease monitoring, as observed in rural Malawi and supported by other literature [30, 31], discouraged patients from seeking health care services, or opted for alternatives, such as using traditional medicine. Consequently, this barrier contributed to delayed care, poorer treatment adherence, and likelihood of poor treatment outcome for this patient population. Furthermore, patients with NCDs did not have the opportunity to benefit from the dissemination of information by peer-patients and patient support groups (as was the case for HIV patients) in Malawi, which given the shortages of health providers and limited patient-provider time are essential interventions [20, 23, 28]. In sum, patients with NCDs and HIV comorbidities in Malawi faced multiple barriers in the acquisition of appropriate knowledge and skills, care and support, in order to successfully manage their conditions.

In the quest of universal health coverage, as part of Malawi’s commitment to the sustainable development goals [1], the affordability and access to NCD services through the public sector presents a formidable challenge, particularly for the rural parts of the country [42]. As a way forward, we recommend further investments in the decentralisation of integrated health services to primary care level, with which Malawi has been able to gain valuable experience over the past decade.

### Strengths and limitations

In efforts to present a spectrum of issues from a heterogenous patient population, details linked to a specific disease may have lacked sufficient representation. However, the diversity in our participants (i.e. patients with multiple chronic conditions) and in the methods used (i.e. survey, interviews, group discussions, and field journal notes) allowed us to present a rich understanding of patient self-management experiences and practices in a resource limited-setting. Further evaluations are needed to assess self-management outcomes and there changes overtime. As our research focused on one rural district in Malawi, the transferability of our findings to contexts with different characteristics, such as an urban district, should be taken into consideration.

## Supporting information

S1 File Research summary. (PDF)

## Abbreviations

AIDS: Acquired immunodeficiency syndrome
CBO: Community-based organisation
CHAM: Christian Health Association of Malawi
CHBC: Community home-based care programmes
FBO: Faith-based organisation
HIV: Human immunodeficiency virus
NCDs: Non-communicable diseases
PHC: Primary health care
sSA: sub-Saharan Africa
