# Supplementary material for: Patients experiences of self-management and strategies for dealing with chronic conditions in rural Malawi
Source: PLoS One. 2018 Jul 2;13(7):e0199977. doi: 10.1371/journal.pone.0199977 (PMC6028088; doi:10.1371/journal.pone.0199977)
Supplement: S1 File — (PDF) [file pone.0199977.s001.pdf]

S1 File. Research summary

Research title: Local models for chronic care and self-management support initiatives in Southern Malawi

Research start date: 1st September 2015
Research end date: 31st August 2018

Investigators and institutional affiliations:
Mrs. Vibian Angwenyi (Principal investigator)[1]
Prof. Joske Bunders-Aelen (Promotor)[2]
Prof. Bart Criel (Promotor)[3]
Prof. Jeffrey V. Lazarus (Promotor)[4]
Dr. Carolien Aantjes (Daily supervisor)[5]
Mr. Murphy Kajumi (Project collaborator)[6]
Prof. Tim Quinlan (Project collaborator)[7]

Qualifications and Institutional affiliations
[1]Doctoral fellow and researcher; Athena Institute, Faculty of Sciences, Vrije Universiteit Amsterdam, The Netherlands; Department of Public Health, Institute of Tropical Medicine (ITM), Antwerp, Belgium; Barcelona Institute for Global Health (ISGlobal), Hospital Clínic, University of Barcelona, Spain
[2]Professor of Biology and Society, Athena Institute, Faculty of Sciences, Vrije Universiteit Amsterdam, The Netherlands
[3]Professor, Department of Public Health, Institute of Tropical Medicine (ITM), Antwerp, Belgium
[4]Professor, Health Systems Research Group, Barcelona Institute for Global Health (ISGlobal), Hospital Clínic, University of Barcelona, Spain
[5]Research fellow; Health Economics & HIV/AIDS Research Division, University of Kwa-Zulu Natal, Durban, South Africa
[6]Project collaborator and Doctoral fellow, Health Economics & HIV/AIDS Research Division, University of Kwa-Zulu Natal, Durban, South Africa
[7]Professor, Health Economics & HIV/AIDS Research Division, University of KwaZulu-Natal, Durban, South Africa

Summary
Health systems in Africa are grappling with considerable challenges including poor health infrastructure, erratic supply chain, inadequate human resources, and lack of pro-poor health financing policies. The double burden of infectious diseases (HIV and tuberculosis) and the rise in non-communicable diseases (hypertension, diabetes and cardiovascular disease) and related mortality, is forcing policy makers to re-orient their health systems to meet the changing health care needs, and develop interventions and services to address this new burden. Most African settings lack clear guidelines and policies to guide prevention and management of chronic conditions. In addition, investments towards chronic diseases have mainly been on secondary and tertiary health care levels, with little/no investments at primary health care (PHC) level where most prevention and health promotion efforts are much needed. While there have been growing calls for integrated services at PHC level, African governments have little to draw on in terms of published evidence base, which contextualises and gives guidance to the concept of integration in resource-constrained settings.

S1 File. Research summary

One approach is to draw from the advancement of HIV/AIDS care programmes in Africa to inform the development of health services for patients with other chronic conditions. Reasons supporting this argument include the transition of HIV care from palliative care to chronic care, which occurred in conjunction with the decentralisation of anti-retroviral treatment (ART) services to primary health care level. In addition, the manifestation of chronic non-communicable diseases (NCDs) and HIV requires similar health care services, hence it is argued there is little justification for them to run parallel to each other in vertical programmes, especially where health systems resources are constrained. Furthermore, there is emerging evidence showing correlation between HIV treatment and early onset of NCDs. Until recently, a few African countries have documented their experiences of integrating HIV community home-based care programme activities into PHC, and their potential to extend health care services to other chronic conditions. Due to the long-term nature of chronic conditions, patients require continuous support and provision of care beyond clinical settings. Hence family members, peers, and community partners play a central role in supporting patients with chronic conditions self-manage their disease.

The overall aim of this research is to explore chronic care interventions at the community and primary health care level, and effectiveness of self-management interventions led by patients, family caregivers, and community partners in community home-based care programmes in Malawi. The specific objectives are:

1. To study the experimentation and learning with chronic care interventions at primary health care and community level. These interventions cover preventive, treatment, care, and self-management aspects.
2. To describe local chronic care models and the interconnections between health care teams, community partners, patients, and families.
3. To assess the perceived effectiveness of self-management interventions in community-based programmes on patients health outcomes.

This study adopts a mixed methods design, consisting of both qualitative and quantitative data collection techniques. The qualitative element of the design is an in-depth case study of CHBC programmes in Phalombe District (Malawi). Overall, data collection includes observations in CHBCs/service delivery points, community mapping, interviews (n=44) and focus group discussions (n=9) with respondents purposively selected at different levels. For instance at primary health care level, engage CHBC programmes run by volunteers in community/faith-based organisations (CBO/FBOs), health workers, family caregivers, and patients living with chronic conditions. At district level, interview government officials in health and social welfare departments, as well as implementing health partners.

The quantitative component is a longitudinal survey nested in the CHBC sites in Phalombe District. Survey inclusion criteria includes any adult patient (18 years and above), with one/more chronic condition, who is newly registered into a CBO/FBO, at point of enrolment to the study. Chronic conditions of interest include non-communicable diseases (e.g. hypertension, diabetes, epilepsy, asthma, stroke, and cancer) and communicable conditions such as HIV. Overall, 140 patients will be recruited across these CBO/FBOs at baseline, and interviews administered at baseline, and repeated after months three, six, and twelve. The outcomes of interest are health status, self-management behaviour and self-efficacy, evaluated using an adapted version of the chronic disease self-management programme measurement scales. We used the following scales, translated and contextualised to the Malawian setting:

S1 File. Research summary

Patient health status scale

i. Self-rated general health measured on a 5-point scale with a higher score (5= "poor health") indicates poorer health.
ii. A 10-point visual numeric scale measured quality of life. Scores ranged from 0 (very poor quality) to 10 (excellent quality), with a higher score indicating better quality of life.
iii. Symptom rating scale i.e. fatigue, pain, shortness of breath, emotional distress, and sleeping difficulties were measured using a visual numeric score (0 = "no symptoms"; 10 = "severe symptoms").
iv. Perceived illness intrusiveness measured using a 4-point scale with a combined score of 9 items. The scale examines the impact of chronic conditions on a patient's daily life i.e. physical well-being and diet, work and finances, family and social relations, recreation and spiritual life.

Self-management health behaviour

i. Communication with physician was measured using a 6-point scale (0 = "never" to 5 = "always") on 9 questions. Examples of questions included "How often do you prepare a list of questions for your doctor?", "How often do you ask questions about the things you want to know", "How often do you ask things you don't understand about your treatment?" etc.
ii. Utilization of health services measured by the number of physician visits, emergency care visits, and number of days/nights hospitalised.
iii. Frequency of exercise/physical activity per week.

Self-efficacy (confidence to manage conditions)

A combined score of 6 items, measured on a 10-point visual numeric scale (1= "no confidence"; 10= "total confidence"). Domains covered include symptom control, emotional functioning, and self-management behaviour.
